# Estimating the prevalence of Non-Verbal Learning Disability (NVLD) from the ABCD sample

**DOI:** 10.1038/s41598-024-58639-x

**Published:** 2024-04-08

**Authors:** Ambra Coccaro, Marie Banich, Irene C. Mammarella, Mario Liotti

**Affiliations:** 1 Department of Developmental Psychology and Socialisation, University of Padova, Padua, Italy; 2 Padova Neuroscience Center, University of Padova, Padua, Italy; 3 Institute of Cognitive Science, University of Colorado, Boulder, USA; 4 Department of Psychology and Neuroscience, University of Colorado, Boulder, USA; 5 Department of Psychology, Simon Fraser University, Burnaby, Canada

**Keywords:** Psychology, Signs and symptoms

## Abstract

Non-Verbal Learning Disability (NVLD) is a neurodevelopmental disorder characterized by deficits in processing visuospatial information but with age-appropriate verbal skills. This cognitive profile has been hypothesized to be associated with atypical white matter, but at the present there is a lack of evidence for this hypothesis. Currently, the condition is not characterized within the main diagnostic systems, in part because no clear set of criteria for characterizing the disorder exists. This report is the first attempt to estimate NVLD prevalence, using two sets of diagnostic criteria, in a large sample of over 11,000 children who were selected without regards to problems of specific nature, either psychological, neurological, physical and/or social. Furthermore, it examined the association between the profile of cognitive abilities and aspects of whole-brain white matter measures in children with and without symptoms associated with NVLD. Participants were drawn from the Adolescent Brain Cognitive Development (ABCD) study, a 10-year longitudinal study of 11,876 children in the U.S. The data used in the present study were drawn from the initial testing point at which the children were 9–10 years old. Prevalence of NVLD based on two distinct sets of criteria, correlations between the measures used to create the criteria, correlations between criteria measures and measures of white matter integrity. The cognitive criteria included measures of visuospatial processing, reading, intelligence and social skills. By varying the cut-offs applied to social skills in conjunction with visuo-spatial difficulties, spared reading skills and intelligence scores, we calculated prevalence for two NVLD groups. White matter characteristics were measures of volume, fractional anisotropy and mean diffusivity. Based on the criteria used, the estimated prevalence of NVLD varied from 1 to 8%. Furthermore, children with NVLD showed a dissociation between measures of visuo-spatial processing not observed in non-NVLD children. At the neurological level, findings provide preliminary evidence of associations between the cognitive profile of NVLD and abnormalities in white matters tracts. The present study documents that exists, within this large non-selected sample, a proportion of youth who show evidence of NVLD. Given those results, it appears essential to establish the best diagnostic criteria, to improve the treatment options and quality of life for children with this disorder.

## Introduction

The term Non-Verbal Learning disability (NVLD) was first introduced by Johnson and Myklebust in 1967^1^: they described children characterized by difficulties in processing information in the non-verbal domain accompanied by spared verbal abilities. In more recent years, several researchers have studied, in more depth, children with visuospatial processing deficits and examined the possible associations with problems concerning attention, motor, academic and social skills, in the absence of frank neurological symptoms or genetic disorders^2,4^. There is evidence showing that the difficulties in visuospatial processing interfere with a child’s quality of social, school or life functioning^5^. In fact, although the core deficits are in visuospatial processing, symptoms can also impact the social domain, especially in relation to non-verbal processing^6^. In particular, children with NVLD show more severe problems in the visuospatial domain compared to either children with Autism Spectrum Disorder (ASD) or Attention Deficit-Hyperactivity Disorder (ADHD)^2,7^. These include difficulties with visuospatial working memory^8,9^, spatial organizational skills and comprehension of spatial descriptions^10,11^, and nonverbal problem-solving abilities^12^, all within the context of preserved language abilities.

At the clinical level, despite increased awareness of the characteristics of NVLD derived from research findings, there are currently no “official” diagnostic criteria for NVLD^13,14^. From a review of the literature, Fisher et al. in 2022^5^ highlighted that the most common criterion used in the past to define NVLD is a discrepancy between verbal and visuospatial intelligence (10 or 15 points between verbal and performance IQ)^13^. However, this criterion has been criticized by some researchers^15,16^, since it is not rare to find such a discrepancy in neurotypical children^17^. Given that NVLD is defined by an impairment in cognitive functioning, more specifically in the realm of visuospatial processing, general heterogeneous consequences for academic achievement and social interactions would be expected. Hence, it could be appropriate not to use the achievement and social measures as a diagnostic criterion. Obviously, which criteria are used (a discrepancy score, or just the level of visuospatial difficulties) will influence estimation of the actual prevalence of NVLD^18^. These considerations have inspired the current investigation with the goal of exploring the prevalence rates of NVLD depending on different criteria for defining this disorder.

At the neurological level, the cognitive profile observed in NVLD has been explained as resulting from a ‘white matter’ syndrome (term coined by Rourke in 1989)^19^, indicating that there are damaged or dysfunctional long myelinated white matter fiber tracts in the brain^20^: these abnormalities have been hypothesized to be mainly located in the right hemisphere. Both animal and human studies point toward the importance of intact white matter for spatial processing^21,22^, but there are no studies in the literature specifically linking white matter to the cognitive profile of NVLD, probably due to the lack of shared diagnostic criteria, which in turn makes it difficult to find appropriate sample sizes for such studies.

The Adolescent Brain Cognitive Development (ABCD) study represents the largest available dataset of children (over 11,000) tested at 9/10 years old, who will be part of the project until 18 years of age. The ABCD database offers researchers an unprecedented opportunity to: (a) test which criteria and cut-offs are most suitable for identifying the characteristics of NVLD; and (b) investigate the white matter contribution to performance in the visuospatial domain in children with a NVLD profile compared to a control group of children of the same age and thus at a similar stage of development. Although not part of the present report, the longitudinal nature of the ABCD study will allow researchers to follow the developmental trajectory of this population and to further confirm or modify the best criteria for identifying NVLD.

### The present study

Given the above considerations, the first goal of the present research was to estimate the prevalence of symptoms associated with a NVLD profile, and to test different criteria in order to investigate which are most informative in describing the population of interest. Surprisingly, considering that the first conceptualization of NVLD was made over 50 years ago, only a few studies have attempted to estimate its prevalence. Moreover, they are generally based on small sample sizes^5^, on non-representative samples in terms of demographic characteristics, and often were drawn from populations with learning disorders (LD) more generally^5^ (but see^23^). A great advantage of the present investigation, compared with the community-drawn sample of Margolis et al.^23^, is that the sample size is larger, it has broad representation of the US in terms of demographics such as socioeconomic and ethnic backgrounds, and it does not involve children/adolescents with selected problems of specific nature, either psychological, neurological, physical and/or social. For these reasons the present research can yield a more accurate estimation of the prevalence of NVLD.

**Figure 1:**
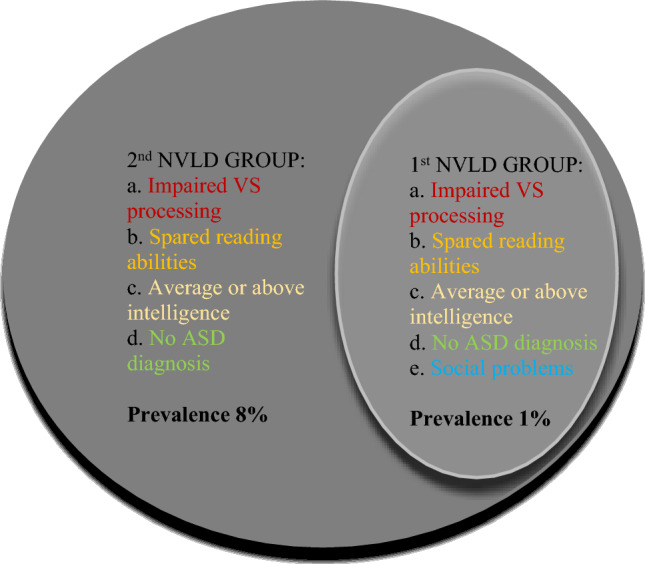
Representation of the NVLD groups, the cognitive criteria, the estimated prevalence and the relation between subgroups [*VS* visuospatial, *ASD* Autism Spectrum Disorder].

The main aim of the present investigation was to estimate the prevalence of the cognitive profile associated with NVLD following two different sets of criteria. Overall requirements for inclusion as NVLD were the presence of visuospatial processing deficits (equal to/below the 16th percentile), preserved reading decoding (above the 25th percentile), intact total or crystallized intelligence (average or above average), and the absence of symptoms of Autistic Spectrum Disorder (ASD). An additional criterion allowed to distinguish two candidate NVLD groups, one with and the other without regards to social problems. In more detail, (1) the first group was characterized by social abilities above the 85th percentile (with social impairment), while (2) the second NVLD group was estimated without using the social impairment as a criterion. See the defining criteria of the two NVLD groups in Fig. 1 and Table 2.

The rationale for estimating the prevalence of the NVLD in the ABCD sample with and without regard to social problems is that the scientific community is still debating about the inclusion of this domain as a criterion, with inconsistences concerning the inclusion of social problems as a defining criterion for NVLD^13^ mainly due to a difficulty in understanding the primary characteristics of NVLD. Moreover, recent evidences are supporting a causal role of the core deficit in the visuospatial domain in explaining the social difficulties^5^. Another consideration is that having these two NVLD groups represents a possibility for future research to have a comparable set of criteria until a consensus on the diagnostic criteria will be reached.

The second aim of the study was to investigate how measures of visuospatial processing correlate with each other and to other criteria used to define a NVLD profile, in the ABCD sample as a whole and likewise in the two identified NVLD groups. The purpose was to determine whether visuo-spatial abilities seem to cohere or dissociate in different manners in the two samples. In addition, we measured sensitivity and specificity of the visuo-spatial tasks used to identify the deficit in the domain of interest in order to provide an estimation of which task has the potential of becoming a diagnostic tool.

The final goal of the research was to shed more light on the neurobiological underpinnings of NVLD by examining whether there are differences in the relationship between the measures of white matter and visuospatial performance in children with and without an NVLD profile. This endeavor may be critical in identifying unique behavioral and neurobiological features of NVLD that may aid in understanding the etiology of the disorder as well as with regards to clinically-relevant considerations, such as diagnosis and the implementation of effective interventions. In order to gain more insight into the NVLD profile, a correlation analysis was applied also between measures of white matter and performances in the other criteria, that is reading abilities, social skills, fluid, crystallized and total intelligence.

## Methods

### Sample

The ABCD dataset (release 3.0; https://abcdstudy.org/) includes 11,878 children aged 9–10 years. This is a 10-year longitudinal dataset being collected at 21 sites across the US. Full recruitment details are described in^24^. It is worth mentioning that participants were drawn from a diverse range of geographic, socioeconomic, ethnic, and health backgrounds meant to be generally similar to that of the US population of 9–10 years old at the initial time point of the study^25,26^.

Institutional review board approval was obtained for each site before data collection and all parents provided written informed consent in addition to assent from the child participants.

### Behavioural measures for the estimation of the prevalence

The following table (Table 1) presents the tasks used as a criterion in the areas of visuospatial processing, intelligence, reading and social skills.

**Table 1:** Domains, tasks and cognitive processes examined in the current study.

Domain	Task	Cognitive process
Visuospatial	Little Man	Mental rotation^27^
	Matrix reasoning (WISC-V)	Visual processing, abstract and spatial perception^28^
	0-back	Memory/recognition of visuospatial stimuli (faces and places). See^29^ for a detailed description
Crystallized intelligence^a^	Picture vocabulary	Language skills and verbal intelligence. See^30^ for a detailed description
	Oral reading recognition	Reading single words^31^
Fluid intelligence^a^	Pattern comparison processing speed	Rapid visual processing^32^
	List sorting working memory	Working memory for sequence stimuli^33^
	Picture sequence memory	Episodic memory^34^
	Flanker	Cognitive flexibility (a variant of^35^)
	Dimensional change card sort	Conflict monitoring abilities^36^
Reading	Oral reading recognition^b^	Reading single words^31^
Social	Child behaviour checklist (CBCL)-Social scale^c^	Social problems^37^

Each row represents the domain with associated cognitive test, and measured cognitive processes, used as criteria in order to identify each NVLD group.

^a^These scores are derived from NIH Toolbox Cognition Battery which has been validated by Heaton et al.^38^ and Akshoomoff et al.^39^ as a means to measure intelligence. All the composite scores on intelligence were calculated by averaging the normalized scaled scores for the relevant test measures (i.e., two for crystallized, five for fluid, and seven for total intelligence composites) and they were extracted from the DEAP (data exploration and analysis) portal offered by the ABCD consortium.

^b^The Reading task (which is one of the two tests of crystallized intelligence) was also used as an independent criterion because it loads less on visuospatial skills compared to Picture Vocabulary.

^c^Social problems were assessed through the Child Behaviour Checklist (CBCL) also known as Achenbach System of Empirically Based Assessment^37^ which is the only questionnaire used as a criterion. It comprises 113 items that measure different behavioral characteristics of the child in the past 6 months, such as “acts too young”, “too dependent”, “doesn’t get along with peers”. We focused only on the social problem scale, measuring social competencies of the child in various contexts.

From a parent self-report questionnaire, we have extracted the information about the diagnosis of ASD.

The choice of including the three visuospatial tasks was made because: (a) there are not known diagnostic indices to measure the core deficit of NVLD; (b) the scientific community did not assess yet if the visuo-spatial deficit is just due to the nature of the material presented, thus involving the perceptual processing (as measured by the matrix reasoning test), or due to the recognition of a visuospatial stimulus (as measured by the 0-back), or the manipulation of it (little man task).

The cut-off (16th and 85th percentile) applied to the visuospatial and the social domain in order to identify the deficits was based on both clinical^39^ and research^23,40^ practices. Instead, the reasoning behind the choice of the cutoffs applied to reading (25th percentile) and intelligence (50th percentile) was based on the evidence that NVLD children have good reading skills^13^ and average or above average total and crystallized intelligence^13^.

### Magnetic resonance imaging

The structural MRI measures used in the present study were the following: one anatomical MRI metric, white matter (WM) volume, and two diffusion MRI metrics: fractional anisotropy (FA) and mean diffusivity (MD). WM volume represents the volume in ${{\text{mm}}}^{3}$. FA measures the directionality of water diffusion within brain tissue that is found to be greater in organized WM tracts. MD refers to the rotationally invariant magnitude of diffusion in the brain and its increase is often reported in case of disease^41^ signaling that pathological processes have affected the structure of white matter and in turn the water motion. Diffusion and anatomical MRI measures were obtained for each hemisphere separately. For a detailed description of the analytic approaches applied to MRI data see^26^.

### Data analysis

The study aimed at estimating the prevalence of the symptoms associated with a NVLD profile and to further explore their neurobiological correlates. First, we set the criteria and identified two different NVLD groups which were differentiated by the cut-offs related to social problems, while keeping constant the criteria applied to visuospatial processing, reading, intelligence and the exclusion of children with a diagnosis of ASD. The 1^st^ NVLD group was estimated with regard to social problems while the 2nd NVLD group was calculated without regard to social problems.

Then we performed three sets of bivariate correlations on the various measures: the first examined the associations of scores between the different behavioral criteria and the second examined the associations between visuospatial performance and white matter measures. The third correlational analysis was applied between white matter measures and the other criteria used to estimate the prevalence of NVLD (reading abilities, social skills, fluid, crystallized and total intelligence). A comparison was made between each correlation observed for the NVLD group with that of the whole ABCD by applying a Z test (Eq. 1) of the difference of the Fisher's Z transformed correlations divided by the standard error of the difference. For sample sizes of n and n2, we found the z of the difference between the z transformed correlations divided by the standard error of the difference of two z scores:

1 $${\text{Z}}-\mathrm{Observed }= ({\text{z}}1 -\mathrm{ z}2) / (\mathrm{square root of }[ (1 /\mathrm{ n }- 3) + (1 /\mathrm{ n}2 - 3)]$$

The last set of analysis included the estimation of sensitivity and specificity that in clinical settings is used to estimate the ability of a test to designate individuals with a disease as positive (sensitivity) and the ones without a disease as negative (specificity). This analysis was performed to investigate if there are differences in the sensitivity and the specificity of the three visuospatial tasks. Sensitivity is calculated by dividing the number of estimated true positive (individuals that are found to be NVLD just based of the score of the task considered) and the true positive (number of individuals with the NVLD profile). Specificity is calculated by dividing the estimated true negative (individual without an NVLD profile as defined by the score on the specific task considered) and the true negative (number of individuals without an NVLD profile). This approach was applied only to the NVLD group without regard to social problems (2nd group) since it contains also the sample identified in the group with regard to social problems (1st group). This analysis was performed with the package caret^42^, specifically with the functions called sensitivity and specificity.

Data analysis was performed in R (v 4.1.0).

## Results

### Prevalence of NVLD profile in the ABCD sample

The present research used two different sets of criteria (for scores corresponding to percentiles used see S1 in Supplementary materials) to define groups of children presenting symptoms associated with the NVLD profile, as shown in Table 2.

**Table 2:** Cut-offs applied to each domain used as criteria in order to identify NVLD groups.

Group	Visuospatial	Reading	Intelligence	Social	ASD
1st NVLD group with social problems and average or above intelligence	≤ 16th percentile on either of the following tasks: Little Man, matrix reasoning or 0-back	≥ 25th percentile on words reading abilities, as measured by oral reading recognition task	≥ 50th percentile on total or crystallized intelligence^a^	≥ 85th percentile on the social subscale of the Child Behavior Checklist	No diagnosis of autism spectrum disorder
2nd NVLD group without regard to social problems and average or above intelligence	≤ 16th percentile on either of the following tasks: Little Man, matrix reasoning or 0-back	≥ 25th percentile in words reading abilities, as measured by oral reading recognition task	≥ 50th percentile on total or crystallized intelligence^a^	Not considered as a criterion	No diagnosis of autism spectrum disorder

^a^Total intelligence is a composite score based on crystallized (measured with picture vocabulary and oral reading recognition test) and fluid (including scores on the pattern comparison processing speed test, the list sorting working memory test, the picture sequence memory test, the Flanker task, and the dimensional change card sort task) components.

Following these different sets of criteria, the estimated sample sizes of the two groups (Fig. 1) were:

Group 1: 144 children (1.21%),

Group 2: 977 children (8.23%).

### Behavioural data

#### Correlations

The second goal of the present research was to investigate how the measures of visuospatial processing correlated to each other and to other criteria used to select the NVLD profile, in the whole population and in the two identified NVLD groups (Table 3).

**Table 3:** The bivariate correlations between the criteria measures used for estimating NVLD symptoms along with the fluid component of.

A								
Matrix reasoning test	0.45***	0.40***	0.34***	− 0.12***	0.33***	0.27***	0.28***	
Little Man task	0.38***	0.31***	0.33***	− 0.12***	0.30***	0.22***		
0-Back task	0.37***	0.33***	0.30***	− 0.12***	0.27***			
Reading test	0.74***	0.88***	0.35***	− 0.12***				
Social problem (CBCL)	− 0.17***	− 0.13***	− 0.16***					
Fluid intelligence	0.83***	0.40***						
Crystallized intelligence	0.85***							
Total intelligence								
	Total intelligence	Crystallized intelligence	Fluid intelligence	Social problem (CBCL)	Reading test	0-Back task	Little Man task	Matrix reasoning test
B								
Matrix reasoning test	0.28**	0.26**	0.17	0.01	0.17	0.03	− 0.20*	
Little Man task	− 0.11	− 0.08	− 0.08	− 0.08	− 0.04	− 0.18*		***
0-Back task	0.02	0.04	0.00	− 0.01	0.03		***	**
Reading test	0.45***	0.74***	0.06	− 0.13		***	***	*
Social problem (CBCL)	− 0.23**	− 0.18*	− 0.18*					
Fluid intelligence	0.84***	0.11					***	
Crystallized intelligence	0.63***		***				***	
Total intelligence							***	
	Total intelligence	Crystallized intelligence	Fluid intelligence	Social problem (CBCL)	Reading test	0-Back task	Little Man task	Matrix reasoning test
C								
Matrix reasoning test	0.19***	0.17***	0.11***	− 0.04	0.10**	− 0.07*	− 0.24***	
Little Man task	0.06	− 0.00	0.07*	− 0.07*	0.05	− 0.19***		***
0-Back task	0.12***	0.05	0.12***	0.00	− 0.03		***	***
Reading test	0.48***	0.77***	0.02	− 0.07*		***	***	***
Social problem (CBCL)	− 0.08*	− 0.07*	− 0.05					*
Fluid intelligence	0.80***	0.02					***	
Crystallized intelligence	0.62***		***				***	
Total intelligence							***	
	Total intelligence	Crystallized intelligence	Fluid intelligence	Social problem (CBCL)	Reading test	0-Back task	Little Man task	Matrix reasoning test

In the upper triangle, there are the correlation coefficients and the associated p values, whereas the lower triangle displayed the significant p value of the z-tests’ result comparing the whole sample to each group. Panel A includes the whole ABCD sample; Panel B presents the NVLD profile with social problems and the most restricted criteria for reading skills and intelligence; Panel C corresponds to the NVLD group without regard to social problems as a criterion but with the strictest cut-off for reading skills and intelligence.

*p < 0.05, **p < 0.01, ***p < 0.001.

The main characteristic that differentiated the NVLD groups from the whole sample was a negative correlation between the mental rotation task and the other two visuospatial tasks, that is matrix reasoning and 0-back. In contrast, in the whole ABCD sample a significant positive correlation was evident between these two tasks and the mental rotation task, consistent with the idea that they measure similar underlying processes related to the visuospatial domain. The NVLD group without regard to social problems was also characterized by a significant negative correlation between the performance on the 0-back task and the scores on the matrix reasoning test that was not present in the whole ABCD sample. Furthermore, the two NVLD groups presented significantly different correlations between scores on the visuospatial tasks compared to the whole sample (as shown in the lower triangle of Table 3; see S2 in Supplementary materials).

Positive correlations between reading skills and the matrix reasoning test were significant in the NVLD group without regard to social problems (group 2), mimicking the results in the overall ABCD sample. Results from the Z-tests highlighted that the two NVLD groups had significantly different correlations between reading and visuospatial abilities compared to the whole ABCD population (see S2 in Supplementary materials for details on statistics).

The scale on social problems extracted from the CBCL (the higher the score, the more pronounced the social problems) was the only measure expected to always be negatively correlated with all the other scores. This held true for the whole ABCD sample. While in the NVLD group without regard to social problems (group 2) we found a significant negative correlation between social problems and the scores on the mental rotation task, that was not found in the other NVLD group in which we used as a criterion social problems on the social subscale of the CBCL. Peculiar to the group 2 were the correlations between visuospatial abilities (measured by matrix reasoning test and 0-back task) and social problems, which were significantly different from the whole sample (see S2 in Supplementary materials for statistics).

There was a positive correlation between the scores on matrix reasoning and total/crystallized intelligence in all the three NVLD groups. In addition, in group 2 there was a significant positive correlation between the fluid component and matrix reasoning, and between total intelligence and the performance on the 0-back task. Furthermore, the same NVLD group without regard to social problems (group 2) presented also a positive correlation between 0-back scores and fluid intelligence, and between the mental rotation task and fluid intelligence. Results of Z-tests indicated that the correlations between performance on thelittle man task and both components of intelligence (in addition to the total score of intelligence) were significantly different between the NVLD groups and the whole ABCD sample (see S2 in Supplementary materials for statistics).

Finally, an unexpected result was found with regards to the correlation between the two components of intelligence within the NVLD groups. It emerged that in the two NVLD groups, the two components of intelligence did not correlate with each other (see S3 in Supplementary materials for statistics).

#### Sensitivity and specificity of visuo-spatial measures

The sensitivity and specificity were calculated for all the visuospatial tasks considered as a criterion and the following table (Table 4) shows the result of the analysis. These results are presented as percentage of correctly identified sample with an NVLD profile (sensitivity) and percentage of the correctly identified sample without the NVLD profile (specificity).

**Table 4:** Sensitivity and specificity of the visuospatial tasks.

	Sensitivity (%)	Specificity (%)
Little man	59	80
Matrix reasoning	53	80
0-back	36	81

The table presents the result of the analysis on the little man, matrix reasoning and 0-back.

The analysis highlight that the sensitivity of the little man task is higher than the matrix reasoning and the 0-back task, while all of them have comparable specificity (around 80%).

### Correlations between white matter and visuospatial performance

This set of correlational analyses was conducted on white matter measures and performance on visuospatial tasks (Table 5).

**Table 5:** The bivariate correlations between white matter measures and visuo-spatial abilities in the different groups.

A									
Matrix reasoning test	0.11***	0.11***	− 0.00	− 0.01	0.02*	0.02	0.27***	0.28***	
Little Man task	0.17***	0.17***	− 0.06***	− 0.06***	0.06***	0.06***	0.22***		
0-Back task	0.13***	0.13***	− 0.01	− 0.01	0.06***	0.06***			
FA LH	0.08***	0.08***	− 0.37***	− 0.41***	0.93***				
FA RH	0.07***	0.07***	− 0.40***	− 0.41***					
MD LH	0.06***	0.07***	0.93***						
MD RH	0.07***	0.07***							
WM vol LH	0.99***								
WM vol RH									
	WM vol RH	WM vol LH	MD RH	MD LH	FA RH	FA LH	0-Back task	Little Man task	Matrix reasoning test
B									
Matrix reasoning test	0.04	0.03	− 0.03	− 0.04	0.06	0.03	0.03	− 0.20*	
Little Man task	0.07	0.07	− 0.18*	− 0.10	− 0.13	0.09	− 0.18*		
0-Back task	− 0.12	− 0.12	0.03	0.00	− 0.03	− 0.04			
FA LH	0.19*	0.19*	− 0.51***	− 0.58***	0.93***				
FA RH	0.17	0.16	− 0.52***	− 0.55***					
MD LH	0.02	0.01	0.93***						
MD RH	0.01	0.00							
WM vol LH	1.00***						**		
WM vol RH							**		
	WM vol RH	WM vol LH	MD RH	MD LH	FA RH	FA LH	0-Back task	Little Man task	Matrix reasoning test
C									
Matrix reasoning test	0.01	0.01	0.01	− 0.02	− 0.02	− 0.01	− 0.07*	− 0.24***	
Little Man task	0.06	0.05	− 0.05	− 0.03	0.02	0.01	− 0.19***		
0-Back task	− 0.02	− 0.02	− 0.04	− 0.03	0.06	0.07			
FA LH	0.07*	0.07	− 0.40***	− 0.45***	0.92***				
FA RH	0.05	0.05	− 0.43***	− 0.43***					
MD LH	0.09**	0.10**	0.91***						
MD RH	0.10**	0.11**							
WM vol LH	1.00***						***	***	*
WM vol RH							***	**	**
	WM vol RH	WM vol LH	MD RH	MD LH	FA RH	FA LH	0-Back task	Little Man task	Matrix reasoning test

The upper triangle displays the correlation coefficients and the associated p values, whereas the lower triangle displays the p-value of the z-tests’ result comparing the whole sample to each group when significant. Panel A presents the correlation matrix between cognitive performances and white matter measures within the whole ABCD sample. Panels B and C correspond to NVLD groups 1 and 2 respectively [*FA* fractional anisotropy, *MD* mean diffusivity, *WM* *vol* White Matter volume].

*p < 0.05, **p < 0.01, ***p < 0.001.

In the whole ABCD sample, a significant positive correlation was found between visuospatial processing, as measured by the Little Man task and the 0-back task, and two white matter indices: volume and fractional anisotropy of both hemispheres. A negative correlation was, instead, found between MD and the performance on the Little Man task. The score on the matrix reasoning test was found to be positively correlated with the volume of white matter in both hemispheres and with the fractional anisotropy of the white matter in the right hemisphere.

For the NVLD group 1, performance on the Little Man task was negatively correlated with mean diffusivity of white matter in the right hemisphere. In contrast, no significant correlations were found for NVLD group 2.

A consistent result found in group 2 was between WM Volume in both hemispheres and performance in the three visuospatial tasks: Z-tests indicated significantly different correlations in the NVLD group compared to the ABCD sample. For groups 1 with the social criterion but only for one specific visuospatial task, that is 0-back task. See Supplementary materials for results of Z-test: S3 and S4 respectively for right and left hemispheres).

### Correlations between white matter and other cognitive criteria: reading, intelligence and social problems

In order to gain more information about the NVLD profile and its differences compared with the all ABCD sample, a correlational analysis was applied between white matter measures and the other criteria used to estimate the prevalence of NVLD: reading skills, social abilities, fluid, crystallized, and total intelligence. Results are shown in Table 6.

**Table 6:**
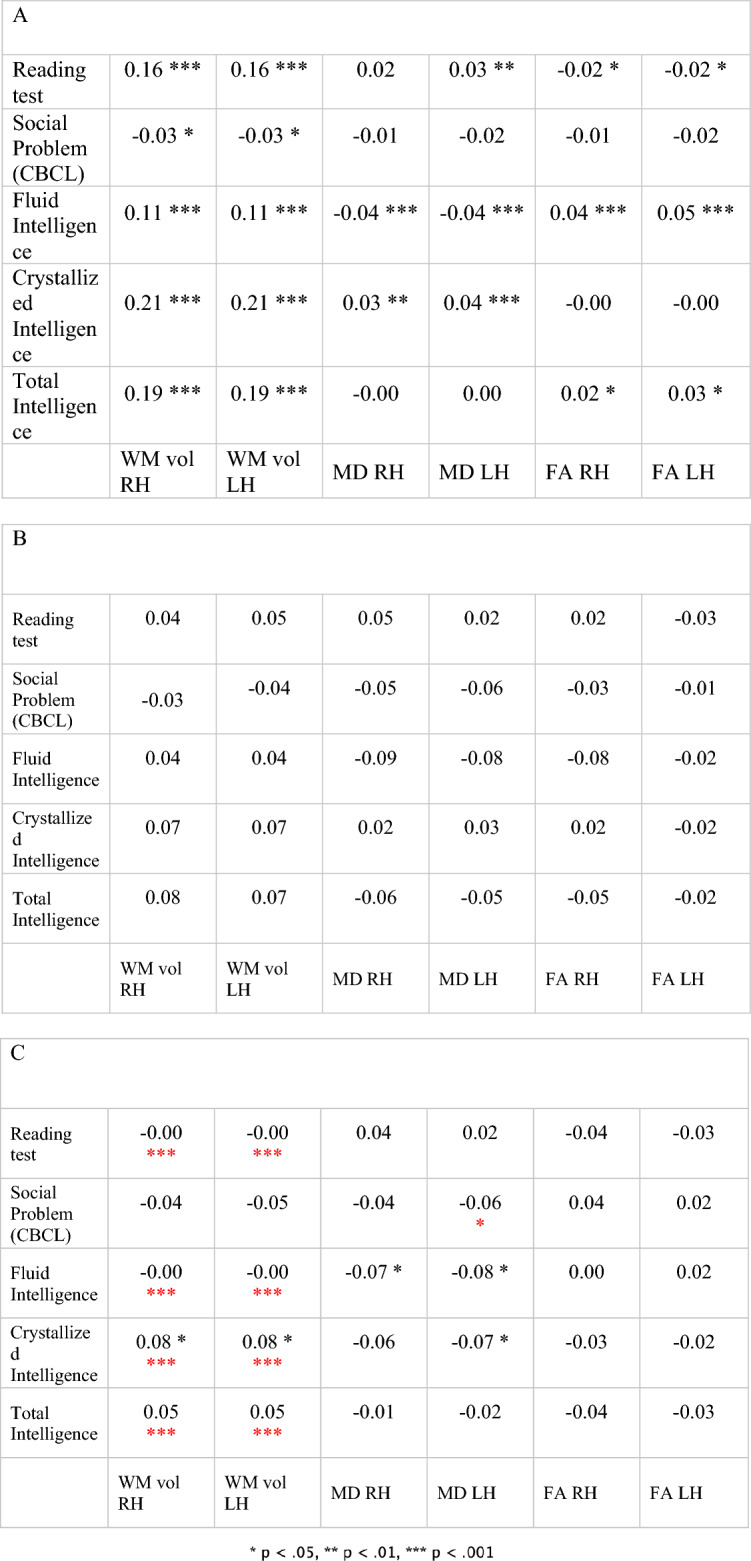
The bivariate correlations between white matter measures and the other criteria used to identify NVLD groups: reading, social problems, intelligence (fluid, crystallized, and total).

The black asterisk indicates a statistically significant correlation, whereas the red asterisk indicates that the z-tests’ result comparing the whole sample to each group are significant. Panel A presents the correlation matrix between cognitive performances and white matter measures within the whole ABCD sample. Panels B and C correspond to NVLD groups 1 and 2 respectively [*FA* fractional anisotropy, *MD* mean diffusivity, *WM* *vol* White Matter volume].

*p < 0.05, **p < 0.01, ***p < 0.001.

Compared to the all ABCD sample, only group 2, without regard to social problems, showed a significantly different correlation between volume of both hemispheres and all cognitive indices, except for social problems. Furthermore, the NVLD group 2 and the all ABCD sample showed a different correlation between social problems and mean diffusivity of the left hemisphere. See Supplementary materials for results of Z-test: S5 and S6 respectively for right and left hemispheres).

## Discussion

### Prevalence of the NVLD profile

The first goal of the present investigation was to estimate the prevalence of the NVLD profile in a large, diverse sample of 11,876 North American children aged 9–10 years old. Our multi-pronged approach using two sets of criteria allowed us to identify two groups composed of 1.21% and 8.23% of the total ABCD sample, respectively. Therefore, we can broadly estimate that from 240,000 to 1.9 million children present symptoms associated with NVLD (among 24.5 million 6–11 years-old children estimated in 2019 by the U.S. Census Bureau).

Since group 1 had very strict criteria for both intelligence/reading as well as social problems, it is likely that it did not include all children presenting the overall symptoms associated with NVLD. For instance, from the literature it emerges that social problems are highly variable and not always present/evident, and possibly unspecific to NVLD. In fact, while it has been found that NVLD is associated with increased risk for internalizing psychopathology^43^, another study found that NVLD was not linked to levels of internalizing psychopathology as rated by the parents^44^. In addition, two comprehensive reviews^45,46^ of the literature highlighted that the results on socioemotional functioning in children with NVLD have been inconsistent.

Regarding group 2, it may represent the best definition of NVLD since it is the one better describing this neurodevelopmental condition, i.e., children with a deficit in visuospatial processing, spared verbal intelligence and reading abilities, and with no diagnosis of ASD. The estimation of the prevalence found to be at about 8% is doubled the one found in Margolis et al. (3–4%)^23^.

This difference between the prevalence rate in the current study and that of Margolis et al.^23^, could be due to various factors. First, we used consistent criteria throughout the whole sample while Margolis et al. used various samples and measures due to the involvement of 3 different datasets^23^. Furthermore, in order for our estimation not to be built on the score of single tasks, we also used composite scores for measuring intelligence which are more comprehensive measures of underlying cognitive processes. Second, Margolis et al. employed the discrepancy between verbal and visuospatial measures, whereas we decided not to include it since this approach has been criticized^15,16^ and it could influence the estimation of the prevalence^18^. Third, even considering that Margolis et al. used inflation factors weights to account for overrepresentation of the psychiatric disorders in their sample, our estimation was made on the ABCD dataset which not only involved mainly neurotypical children but it is composed by a sample that is more representative of the whole population in terms of geographic, socioeconomic and ethnic backgrounds than that of Margolis et al. Finally, the sample size of the ABCD dataset is considerably larger than the one of Margolis et al. (11 878 vs. 2 596).

### Cognitive profile of NVLD

#### Social and visuospatial domains

The inclusion of social problems as a criterion to estimate the prevalence of NVLD seems to identify a restricted portion of children having deficits in nonverbal abilities. In contrast to Rourke’s cognitive model of NVLD^19^, recent research highlighted that social problems, while not a primary feature, may still be associated with the disorder^13^. However, we did not find consistent results on the link between visuospatial abilities and social problems, possibly indicating that there is not a tight association between the two in children with NVLD, even if social problems are used as a criterion. For this reason, we believe that the cut-off criterion of the second group better captures the clinical group of interest, since social problems are not always present and the thresholds imposed to intelligence, reading and visuospatial abilities are likely to reveal the actual difficulties that a child with NVLD has to face.

Among the identified groups presenting the main symptoms associated with NVLD, we found an interesting result that should be taken into account when measuring the performance in the visuospatial domain. The performance in a strictly visuospatial task involving active information manipulation, as in the little an test, should be employed along with other visuospatial tasks. In fact, only the NVLD groups showed a negative correlation between performance on the little man task and the other two visuospatial tests, i.e., matrix reasoning and 0-back tasks. A possible explanation is that NVLD children may try to compensate for their visuospatial deficits with their intact verbal reasoning abilities. Such compensation likely works better for the matrix reasoning and the 0-back task, which mainly involve abstract reasoning, memory and attention, than for the little man task, which likely relies on strategies strictly related to the visuospatial domain^47,48^. Furthermore, in the NVLD groups, intelligence is correlated with visuospatial performances in all visuospatial tasks except for the little man task, possibly indicating that they cannot use their intact cognitive abilities to perform this task.

##### Sensitivity and specificity of visuo-spatial tasks

The analysis on sensitivity and specificity of the tasks in the visuo-spatial domain revealed an interesting gap between the visuo-spatial tasks. While there were no differences in the specificity (around 80%), the sensitivity of the little man task (59%) was higher than matrix reasoning (53%) and the 0-back (36%). These results highlight that while all the tasks can accurately identify individuals not having an NVLD profile, only the little man task is sensitive at identifying individuals with an NVLD profile 59% of the time. It should be noted that since none of the tasks are designed to be a normative neuropsychological test. Achieving a sensitivity of 59% makes a promising starting point for developing test tailored to measure the visuo-spatial deficits mainly observed in NVLD.

#### Intelligence

The two NVLD groups did not show the positive correlation between crystallized and fluid intelligences found in the whole ABCD sample and in the other NVLD group. In fact, this finding indicates that these two components of intelligence are likely independent. It should be noted that theories of intelligence^48^ assume that the two components develop by mutual interactions and this reciprocal relation is hypothesized to be beneficial for both. Since this interactive process is likely not present in NVLD children, it would be useful to understand how this uncoupling is linked to development of processes such as reading and mathematics. In fact, a recent metanalysis^49^ highlighted that fluid intelligence and reading/mathematics are able to predict each other over developmental phases. Yet we did not find any significant correlation between reading and fluid intelligence in NVLD groups.

The lack of association between these two facets of intelligence might be helpful as an alternative to using a discrepancy score (generally 10–15 points)^13^ between verbal and performance IQ in order to study groups with and without a NVLD profile. For instance, by looking at their developmental trajectories, in future studies one could investigate if the lack of correlation between the two components of intelligence is a stable characteristic of this population.

### Brain-behaviour linkages

As predicted, associations were found between the NVLD profile and aspects of white matter connectivity in the brain. In NVLD groups 1 (in which social problems serve as a criteria), the lower the performance in visuospatial processing, as measured by the little man task, the higher the mean diffusivity in the right hemisphere. This result indicated that in NVLD a greater disorganization of white matter tracts in the right hemisphere is linked to worse performance in mentally rotating visuospatial materials. Note however that this finding was not replicated in NVLD group 2 and did not extend to the other two tasks measuring visuospatial processing.

When analyzing the differences between correlations in the NVLD groups compared to the whole ABCD sample, the relationship of the visuospatial processing with the volume of the white matter yielded interesting results. In the NVLD group without regard to social problems (i.e., group 2), there was no relationship between visuospatial ability, as measured by all three tasks, and the volume of white matter. This link was significantly different compared to the whole population, in whom the higher the volume, the better the visuospatial performance. In the other NVLD group (i.e., group 1) there were relationships with white matter volume but only for one spatial task, the 0-back task.

Concerning the correlations between white matter measures and social problems, the group without regard to social problems (group 2) showed an interesting result compared to the all ABCD sample. In fact, while the direction of the correlation was negative in both samples, only in the NVLD group we found a significant correlation. The lower the mean diffusivity in the left hemisphere, the greater were the problems in the social domain.

In conclusion, the most relevant finding for the NVLD profile (group 2) in relation to visuospatial performance and white matter measures appeared to be the absence of the significant association found in typically developing children, for whom non-verbal IQ and visuospatial abilities were associated with white matter integrity. We propose that this lack of association may provide an important information regarding the neurobiological basis of the impairments found in NVLD^22^. This finding represents a first step toward a more sophisticated analysis to model this relationship between white matter and visuo-spatial ability^50^ and to better test the hypothesis of the relationship between the two^20^.

## Limitations and future directions

The current study is not without limitations. While the sample size was very large, the measurement of visuo-spatial processing skills was restricted, due to the general scope of the ABCD study to test a wide variety of abilities and multiple factors that might influence brain development. In particular, we were not able to include measures assessing visuo-constructive, visuomotor and fine motor abilities which are often used in assessments for NVLD. Therefore, our estimation of 8% (977 children) prevalence of children with a likely diagnosis of NVLD should be taken with caution, while nonetheless pointing to the fact that it will be important to do future research to deepen our knowledge of this neurodevelopmental disorder.

A future goal of this line of research may be to follow the developmental trajectories of children with the NVLD profile in order to investigate how coping strategies may evolve as they grow up, and to find out whether cut-offs and tasks utilized in the present investigation continue to be the best at identifying children with NVLD in transition from middle childhood to the pre-adolescent and adolescent phase. Furthermore, the enormous heterogeneity of the measures present in ABCD protocol will allow researchers to apply other approaches to the study of brain correlates of the NVLD profile, for instance by focusing on specific regions and on other techniques, such as connectivity patterns in networks of interests. Finally, another future direction could be to investigate other weaknesses and strengths that are peculiar of the sample presenting an NVLD profile.

## Conclusions

The above limitations notwithstanding, the present research represents the first attempt to estimate the prevalence of NVLD in a large sample of typically developing children. We found that depending on the criteria, the estimated incidence of the profile varied from 1 to 8%. Unlike the ABCD sample as a whole, the NVLD groups showed uncorrelated or negatively correlated performances in the three tasks measuring visuospatial performance indicating that they may apply different strategies to compensate the deficit depending on the demands of the task using visuospatial materials. Moreover, we found that while in typically developing children higher volume of white matter tracts was associated with better visuospatial abilities, children with NVLD did not show this link, bringing support to the notion that an atypical mechanism involving the myelinated tracts, particularly in the right hemisphere, could help to explain the cognitive profile of NVLD.

## Data and availability

Data used in the preparation of this article were obtained from the Adolescent Brain Cognitive Development (ABCD) Study (https://abcdstudy.org), held in the NIMH Data Archive (NDA).

### Supplementary Information

Supplementary Information.
