# Supplementary material for: Estimating the prevalence of Non-Verbal Learning Disability (NVLD) from the ABCD sample
Source: Sci Rep. 2024 Apr 8;14:8212. doi: 10.1038/s41598-024-58639-x (PMC11001940; doi:10.1038/s41598-024-58639-x)
Supplement: Supplementary file 1 — Supplementary Information. [file 41598_2024_58639_MOESM1_ESM.docx]

## Supplementary materials

**Table S1.** Cut-offs applied to cognitive tasks used as criteria for identifying NVLD profiles.

| **VISUOSPATIAL DOMAIN** |  |  |
| --- | --- | --- |
| *Little Man* | 16th%ile | 0.44 |
| *Matrix Reasoning* | 16th%ile | 7 |
| *0-Back* | 16th%ile | 0.66 |
| **READING SKILLS** |  |  |
| *Oral Reading Recognition* | 25th%ile | 90 |
| **INTELLIGENCE** |  |  |
| *Total Intelligence* | 50th%ile | 100 |
| *Crystallized Intelligence* | 50th%ile | 90 |
| **SOCIAL DOMAIN** |  |  |
| *Child Behavior Checklist - Social subscale* | 85th%ile | 57 |
|  |  |  |

**Table S2.** Results from the Z test comparing the correlations within the cognitive criteria, between each NVLD group and the whole ABCD sample.

| **Correlation** | **Group 1** | **Group 2** |
| --- | --- | --- |
| *Little Man – Matrix* | z = 5.85 | z = 16.07 |
|  | p < .001 *** | p < .001 *** |
| *Little Man – 0-Back* | z = 4.78 | z = 12.54 |
|  | p < .001 *** | p < .001 *** |
| *Matrix – 0-Back* | z = 2.89 | z = 10.42 |
|  | p < .01 ** | p < .001 *** |
| *Little Man – Reading* | z = 4.12 | z = 7.75 |
|  | p < .001 *** | p < .001 *** |
| *Matrix – Reading* | z = 2.13 | z = 7.31 |
|  | p < .05 * | p < .001 *** |
| *0-Back – Reading* | z = 2.87 | z = 9.19 |
|  | p < .01 ** | p < .001 *** |
| *Little Man – Intelligence* | z = 5.97 | z = 10.34 |
|  | p < .001 *** | p < .001 *** |
| *Little Man – Crystallized* | z = 4.72 | z = 9.66 |
|  | p < .001 *** | p < .001 *** |
| *Little Man – Fluid* | z = 5.01 | z = 8.07 |
|  | p < .001 *** | p < .001 *** |
| *Social domain – Little Man* | z = 0.52 | z = 1.50 |
|  | p = 0.60 | p = 0.13 |
| *Social domain – Matrix* | z = 1.46 | z = 2.23 |
|  | p = 0.14 | p < .05 * |
| *Social domain – 0-Back* | z = 1.25 | z = 3.72 |
|  | p = 0.21 | p < .001 *** |
| *Fluid – Crystallized* | z = 3.7 | z = 12.27 |
|  | p < .001 *** | p < .001 *** |

**Table S3.** Results from the Z test comparing the correlations between VS performance and measures of white matter for the right hemisphere in each NVLD group and in the whole ABCD sample.

| **Correlation** | **Group 1** | **Group 2** |
| --- | --- | --- |
| *Matrix - WM Volume* | z = 0.75 | z = 2.88 |
|  | p = 0.45 | p < .01 ** |
| *Matrix - Fractional Anisotropy* | z = 0.44 | z = 1.24 |
|  | p = .66 | p = .22 |
| *Matrix - Mean Diffusivity* | z = 0.28 | z = 0.36 |
|  | p = .78 | p = .72 |
| *Little Man - WM Volume* | z = 1.22 | z = 3.56 |
|  | p = 0.22 | p < .01 ** |
| *Little Man - Fractional Anisotropy* | z = 0.72 | z = 1.47 |
|  | p = .47 | p = .14 |
| *Little Man - Mean Diffusivity* | z = 1.49 | z = 0.24 |
|  | p = .14 | p = .81 |
| *0-Back - WM Volume* | z = 3.08 | z = 4.70 |
|  | p < .01 ** | p < .001 *** |
| *0-Back - Fractional Anisotropy* | z = 1.02 | z = 0.18 |
|  | p = .31 | p = .86 |
| *0-Back - Mean Diffusivity* | z = 0.46 | z = 1.10 |
|  | p = .65 | p = .27 |

**Table S4.** Results from the Z test comparing the correlations between VS performance and measures of white matter for the left hemisphere in each NVLD group and in the whole ABCD sample.

|  | **Group 1** | **Group 2** |
| --- | --- | --- |
| *Matrix - WM Volume* | z = 0.95 | z = 2.96 |
|  | p = 0.34 | p < .05 * |
| *Matrix - Fractional Anisotropy* | z = 0.17 | z = 0.67 |
|  | p = .87 | p = .50 |
| *Matrix - Mean Diffusivity* | z = 0.36 | z = 0.23 |
|  | p = .72 | p = .82 |
| *Little Man - WM Volume* | z = 1.23 | z = 3.65 |
|  | p = 0.22 | p < .001 *** |
| *Little Man - Fractional Anisotropy* | z = 0.29 | z = 1.71 |
|  | p = .77 | p = .09 |
| *Little Man - Mean Diffusivity* | z = 0.53 | z = 0.84 |
|  | p = .60 | p = .40 |
| *0-Back - WM Volume* | z = 3.02 | z = 4.72 |
|  | p < .01 ** | p < .001 *** |
| *0-Back - Fractional Anisotropy* | z = 1.11 | z = 0.29 |
|  | p = .27 | p = .77 |
| *0-Back - Mean Diffusivity* | z = 0.17 | z = 0.58 |
|  | p = .86 | p = .56 |

**Table S5.** Results from the Z test comparing the correlations between intelligence (total, crystallized and fluid), reading, social problems and measures of white matter for the right hemisphere in each NVLD group and in the whole ABCD sample.

|  | **Group 1** | **Group 2** |
| --- | --- | --- |
| *Reading - WM Volume* | z = 1.34 | z = 4.87 |
|  | p = .18 | p < .001 *** |
| *Reading - Fractional Anisotropy* | z = 0.45 | z = .58 |
|  | p = .65 | p = .56 |
| *Reading - Mean Diffusivity* | z = 0.07 | z = 0.52 |
|  | p = .95 | p = .61 |
| *Fluid Intelligence - WM Volume* | z = 0.90 | z = 3.55 |
|  | p = .37 | p < .001 *** |
| *Fluid Intelligence - Fractional Anisotropy* | z = 1.43 | z = 1.25 |
|  | p = .15 | p = .21 |
| *Fluid Intelligence - Mean Diffusivity* | z = 0.61 | z = 0.80 |
|  | p = .54 | p = .42 |
| *Crystallized Intelligence - WM Volume* | z = 1.62 | z = 3.86 |
|  | p = .11 | p < .001 *** |
| *Crystallized Intelligence - Fractional Anisotropy* | z = 0.28 | z = 0.90 |
|  | p = .78 | p = .37 |
| *Crystallized Intelligence - Mean Diffusivity* | z = 0.12 | z = 0.92 |
|  | p = .91 | p = .36 |
| *Total Intelligence - WM Volume* | z = 1.41 | z = 4.42 |
|  | p = .16 | p < .001 *** |
| *Total Intelligence – Fractional Anisotropy* | z = 0.82 | z = 1.27 |
|  | p = .41 | p = .20 |
| *Total Intelligence – Mean Diffusivity* | z = 0.66 | z = 0.28 |
|  | p = .51 | p = .78 |
| *Social scale - WM Volume* | z = 0.03 | z = 0.30 |
|  | p = .98 | p = .76 |
| *Social scale – Fractional Anisotropy* | z = 0.19 | z = 1.62 |
|  | p = .85 | p = .10 |
| *Social scale- Mean Diffusivity* | z = 0.77 | z = 1.60 |
|  | p = .44 | p = .11 |

**Table S6.** Results from the Z test comparing the correlations between intelligence (total, crystallized and fluid), reading, social problems and measures of white matter for the left hemisphere in each NVLD group and in the whole ABCD sample.

|  | **Group 1** | **Group 2** |
| --- | --- | --- |
| *Reading - WM Volume* | z = 1.31 | z = 4.85 |
|  | p = .19 | p < .001 *** |
| *Reading - Fractional Anisotropy* | z = 0.16 | z = .14 |
|  | p = .88 | p = .89 |
| *Reading - Mean Diffusivity* | z = 0.01 | z = 0.07 |
|  | p = .99 | p = .95 |
| *Fluid Intelligence - WM Volume* | z = 0.92 | z = 3.58 |
|  | p = .36 | p < .001 *** |
| *Fluid Intelligence - Fractional Anisotropy* | z = 0.84 | z = 1.05 |
|  | p = .04 | p = .29 |
| *Fluid Intelligence - Mean Diffusivity* | z = 0.48 | z = 1.17 |
|  | p = .63 | p = .24 |
| *Crystallized Intelligence - WM Volume* | z = 1.63 | z = 4.14 |
|  | p = .10 | p < .001 *** |
| *Crystallized Intelligence - Fractional Anisotropy* | z = 0.22 | z = 0.64 |
|  | p = .83 | p = .53 |
| *Crystallized Intelligence - Mean Diffusivity* | z = 0.11 | z = 0.96 |
|  | p = .91 | p = .34 |
| *Total Intelligence - WM Volume* | z = 1.43 | z = 3.64 |
|  | p = .15 | p < .001 *** |
| *Total Intelligence – Fractional Anisotropy* | z = 0.61 | z = 0.90 |
|  | p = .54 | p = .37 |
| *Total Intelligence – Mean Diffusivity* | z = 0.56 | z = 0.54 |
|  | p = .58 | p = .59 |
| *Social scale - WM Volume* | z = 0.04 | z = 0.35 |
|  | p = .97 | p = .73 |
| *Social scale – Fractional Anisotropy* | z = 0.04 | z = 1.26 |
|  | p = .97 | p = .21 |
| *Social scale- Mean Diffusivity* | z = 0.93 | z = 2.14 |
|  | p = .35 | p < .05 * |
